# A method of calculating the bearing capacity of sand pile composite foundations in a mucky soil layer considering consolidation

**DOI:** 10.1038/s41598-021-95678-0

**Published:** 2021-08-20

**Authors:** Yongtao Zhang, Yuqing Liu, Huiwu Luo, Peishuai Chen, Dejie Li, Enlong Liu, Benliang Yang

**Affiliations:** 1 School of Civil Engineering, Tongji University, Shanghai, 200092 China; 2 CCCC Second Harbor Engineering Company Co., Ltd., Wuhan, 430040 China; 3 College of Water Resources and Hydropower, State Key Laboratory of Hydraulics and Mountain River Engineering, Sichuan University, Chengdu, 610065 China

**Keywords:** Civil engineering, Engineering

## Abstract

In engineering practice, the measured bearing capacity of a sand pile composite foundation in a mucky soil layer is much larger than the design value. Based on the sand pile construction and the load application process, a method of calculating the bearing capacity of the foundation based on the effective stress was proposed. Cavity diameter expansion in sand pile construction was simplified into a planar problem, and the cavity expansion theory was used to establish the expression of the rate of displacement and the horizontal stress increase. Based on the e–p curve and the calculation of the degree of consolidation, the relationships between the horizontal and vertical effective stress and the void ratio were obtained. According to the close relationship between the bearing capacity of the foundation in a mucky soil layer and the water content, an expression describing the relationships between the bearing capacity of the foundation, effective stress, void ratio, and water content was established. For the temporary engineering foundation treatment project, which needs a high bearing capacity but allows large foundation deformation, the design of sand pile composite foundations uses these relationships to take the consolidation effect of mucky soil into consideration, thereby reducing the replacement rate and lowering the construction cost.

## Introduction

Large caissons are widely applied in bridge tower foundations and anchor foundations because they can provide reliable bearing capacity and stability^1^. Since a large caisson has a large dead load, damage or rapid subsidence of an untreated superficial foundation is likely to occur under the dead load of the caisson. Therefore, the original foundation needs to be treated to ensure the smooth construction of the caisson. According to the construction technology of the caisson, the foundation reinforcement material needs to be excavated gradually with the sinking of the caisson, so the sand pile composite foundation is the preferred scheme.

**Figure 1:**
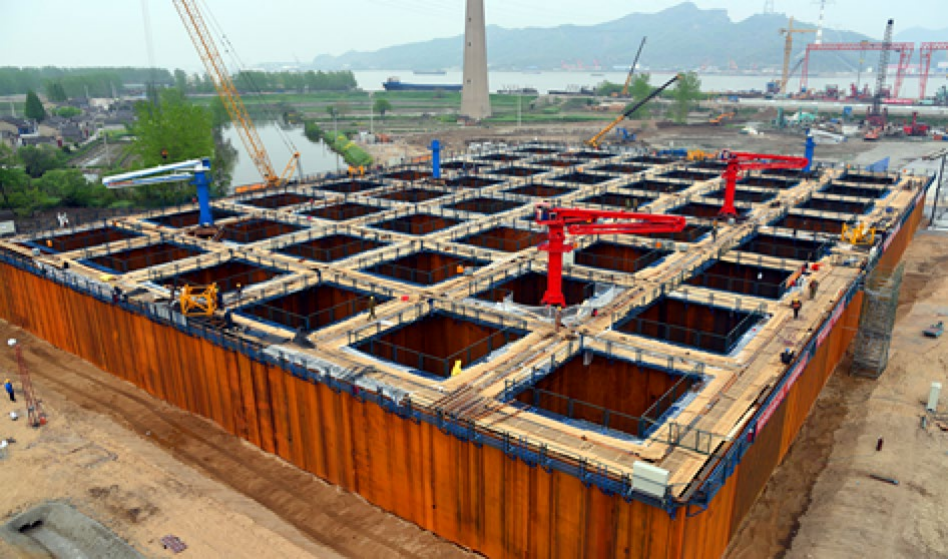
Wufeng mountain Yangtze river bridge’s north anchorage caisson.

The Wufengshan Yangtze River Bridge is part of the Lianzheng railway crossing Yangtze river control project (see Fig. 1). Its north anchorage of the large caisson foundation is the world's largest land open caisson with a length of 100 m and a width of 70 m. A plate load test was carried out before the foundation of the North Anchorage Caisson began to sink, the results of which show that the measured bearing capacity is much larger than the design value. The excessively strong bearing capacity of the foundation not only causes resources to be wasted but also hinders the sinking of the caisson, which leads to more soil support being excavated to increase the large caisson sinking coefficient, increasing the risk of the cracking of the caisson as the support span becomes larger. The foundation treatment of the North Anchorage Caisson of the Wufengshan Yangtze River Bridge is designed according to the requirements of the permanent works. However, the bearing capacity of foundation is too high to prevent the open caisson sinking, which causes the structure stress of the large caisson to exceed the permissible value. Sand pile composite foundations have been used for a long time, and many in-depth researches have been conducted in this field. Two main factors affect the bearing capacity of a sand pile composite foundation: the construction of sand compaction piles and the overburden load. First, the construction of sand compaction piles leads to an increase in the bearing capacity of the soil foundation between the piles. Butterfield^2^ first proposed the use of the cylindrical cavity expansion under plane strain conditions to solve the problem of pile penetration. Vesic^3^ summarized the solution to the expansion of spherical cavities and cylindrical cavities and also extended the cavity expansion theory to compressible soil to obtain the calculation equation of the ultimate bearing capacity of the soil. Randolph^4^ applied the cavity expansion theory and finite element analysis to analyze the stress generated by the piles in the clay and the subsequent consolidation. Yu and Houlsby^5^ used the finite element method and cavity expansion theory to analyze the load of expansive soil. Collins^6^ used the critical state model to derive the large strain analytical solution for undrained cylindrical and spherical cavity expansion. According to the cavity expansion theory and the strain path, Zhou^7^ proposed a method of calculating soil displacement around non-circular piles in clay. Second, the load from the top compacts the soil, increasing the bearing capacity of the foundation. When treating the soft soil foundation using the drainage consolidation method, Barron^8^ and Richart^9^ applied two assumptions, i.e., free strain and equal strain, to solve the radial drainage degree of consolidation of the soil layer, believing that when the drain spacing ratio was greater than five, the average degrees of consolidation of the foundation obtained by the above two calculation methods were very close. Onoue^10^ considered the analytical solution to sand well consolidation with smearing. Olson^11^ and Lekha^12^ proposed analytical solutions to sand well consolidation under variable load conditions. There is a unique relationship between the effective stress and the void ratio of saturated soft clay, and it is independent of the conditions of drainage^13^. The bearing capacity of the foundation is related to the cohesion *c*, the internal friction angle *ϕ*, and the uniform load *q* acting on both sides of the foundation^14^, but the change of the cohesion and the internal friction angle caused by the increase of effective stress remains to be verified.

Current research indicates that sand pile construction and preloading of piles can increase the bearing capacity of the foundation. In engineering applications, foundation settlement tends to be the key indicator of whether the design and the bearing capacity can meet the needs easily. Thus, the improvement of the bearing capacity of a foundation has little effect on the design of foundation treatment. For large caisson constructions, however, the foundation treatment is a temporary work, and the bearing capacity is the key indicator, so foundation settlement is generally paid little attention. The construction of the caisson in the pre-fabrication process takes a relatively long time, which is the process of applying loading step by step. The foundation produces large consolidation deformation because of the additional load and the drainage channel in the sand pile. As a result, the measured bearing capacity of a sand pile composite foundation is much larger than the design value. In this study, the construction and preloading process of sand piles were systematically studied. Moreover, an expression describing the relationships between the bearing capacity of the foundation, effective stress, void ratio, and water content was formulated to optimize the design.

## Increase of foundation stress induced by sand pile construction

Pipe casing is often applied in the construction of sand piles, that is, the lower-end discharge method. During the sand pile construction, a cavity is made through the pipe casing, the lower part of the casing is closed, and the cavity is expanded in the mucky soil layer by mechanical static pressure and vibration. Then, as the casing is lifted, the valve at the bottom of the casing is automatically opened, and the cavity is filled with sand to form the sand pile. This process can be simplified into cavity expansion. In this study, this theory was used to calculate the increase of the bearing capacity of the foundation in the mucky soil layer caused by sand pile construction.

### Cavity expansion theory and basic assumptions

**Figure 2:**
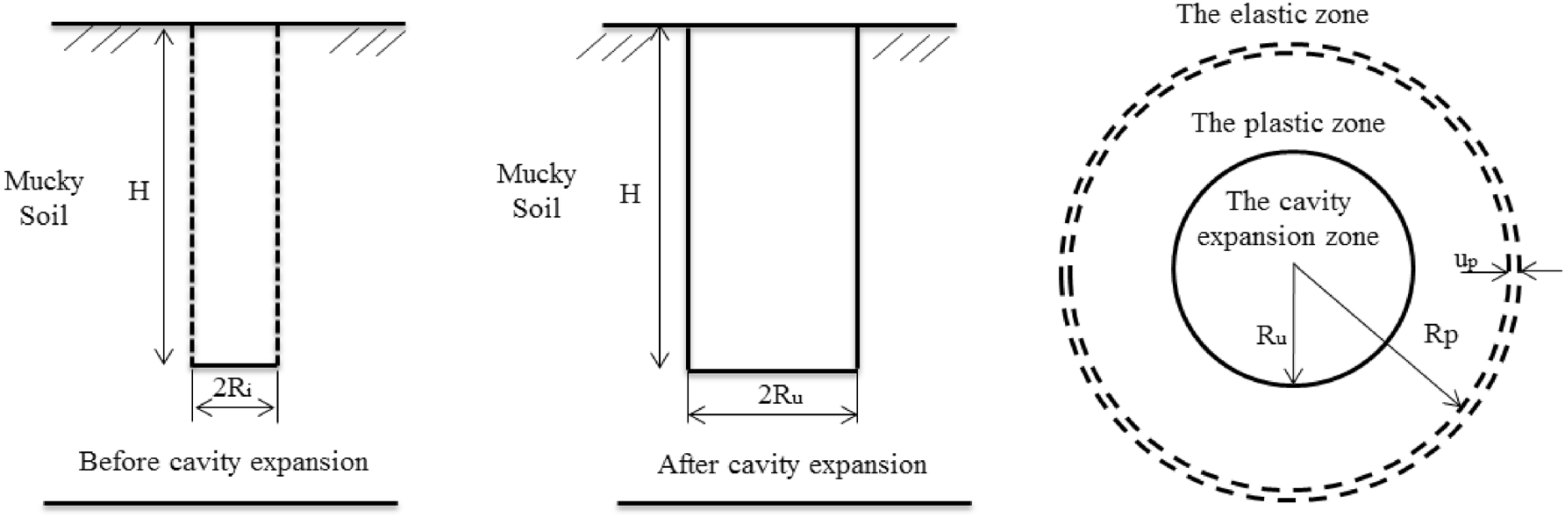
Cavity expansion model.

The completed sand pile was assumed to be ideally cylindrical, and its size fully met the design requirements. The sand pile construction process was carried out as shown in Fig. 2.

The analysis of the cavity expansion theory^3^ was based on the following assumptions: (1) the soil mass is an ideal, homogeneous, and isotropic elastic plastic body; (2) the small cavity expands in an infinite soil mass; (3) the soil yield criterion is the Mohr–Coulomb yield criterion; (4) the soil pressure of the cavity wall is static before the expansion; and (5) the sand pile is made of pure sand without cohesive force, and the yield deformation is not considered.

### Basic equations

The radial stress of the soil around the pile was denoted by $\sigma_{r}$, the circumferential stress was denoted by $\sigma_{\theta }$, and the sand pile construction process was simplified into a problem of plane strain axial symmetry. The polar coordinates were used without considering the initial stress field, and the equilibrium differential equation was obtained as follows:

1 $$\frac{{d\sigma_{r} }}{dr} + \frac{{\sigma_{r} - \sigma_{\theta } }}{r} = 0.$$

Geometric equation is

2 $$\varepsilon_{r} = \frac{{du_{r} }}{dr}.$$

In the elastic deformation phase, the stress function $\psi$ was assumed to be only a function of the radial coordinate *r*:

3 $$\psi = L\ln r,$$

where $r$ is the radial coordinate, and L represents the boundary constant.

During the plastic deformation stage, the parameters are selected as consolidated undrained parameters, the Mohr–Coulomb yield criterion was used:

4 $$(\sigma_{r} - \sigma_{\theta } ) = (\sigma_{r} + \sigma_{\theta } )\sin \varphi + 2c\cos \varphi .$$

### Solution of displacement field and stress field in the surrounding soil layer

Assuming that the problem is an elastic deformation in a plastic problem^15^. In the elastic zone, the stress and strain were defined by the following equations:

5 $$\varepsilon_{r} = \frac{{1 - v^{2} }}{E}\left( {\sigma_{r} - \frac{v}{1 - v}\sigma_{\theta } } \right),$$

6 $$\sigma_{r} + \sigma_{\theta } = 0,$$

7 $$\sigma_{r} = - \sigma_{\theta } = \frac{{R_{i}^{2} p}}{{r^{2} }}.$$

The displacement of the soil around the pile should satisfied the following equation:

8 $$u = \frac{{(1 + v)R_{i}^{2} }}{E}\frac{p}{r} = \frac{(1 + v)}{E}r\sigma_{r} ,$$

where $r$ is the radial coordinate, $u_{r}$ is the radial displacement, $R_{i}$ is the initial radius of the cavity, $p$ is the initial radial stress, *E* is the elastic modulus, and $v$ is Poisson's ratio.

Based on Eqs. (4) and (1), the following equation was obtained by solving the equilibrium differential equation:

9 $$\sigma_{r} = (p_{u} + Cctg\varphi )\left( {\frac{{R_{u} }}{r}} \right)^{{\frac{2\sin \varphi }{{1 + \sin \varphi }}}} - Cctg\varphi .$$

By satisfying Eqs. (4) and (6) under the common boundary conditions of elasticity and plasticity, the following equation was obtained:

10 $$\sigma_{p} = \sigma_{r} = C\cos \varphi .$$

On the boundary between the elastic zone and the plastic zone, the displacement of the overall expansion of the plastic zone was obtained on the basis of Eq. (8):

11 $$u_{p} = \frac{(1 + v)}{E}R_{p} \sigma_{p} .$$

The radial stress near the boundary of the plastic zone was expressed as follows:

12 $$\sigma_{p} = (p_{u} + Cctg\varphi )\left( {\frac{{R_{u} }}{{R_{p} }}} \right)^{{\frac{2\sin \varphi }{{1 + \sin \varphi }}}} - Cctg\varphi .$$

Based on Eqs. (10) and (12), the equation for the pressure inside the cylindrical cavity was obtained:

13 $$p_{u} = C\left[ {\frac{\cos \varphi + ctg\varphi }{{\left( {\frac{{R_{u} }}{{R_{p} }}} \right)^{{\frac{2\sin \varphi }{{1 + \sin \varphi }}}} }} - ctg\varphi } \right].$$

The volume change after the expansion of the cylindrical cavity is equal to the volume change of the elastic zone and that of the plastic zone, and the following equation was obtained:

14 $$\pi R_{u}^{2} - \pi R_{i}^{2} = \pi R_{p}^{2} - \pi (R_{p} - u_{p} )^{2} + \pi (R_{p}^{2} - R_{u}^{2} )\Delta ,$$

where $R_{p}$ is the maximum radius of the plastic zone, $R_{u}$ is the final radius of the cavity, $u_{p}$ is the overall displacement of the plastic zone, and $\Delta$ is the average volumetric strain of the plastic zone.

It was considered that the radius of the initial cavity approaches zero, so it was simplified as $R_{i}^{2} \to 0$, and the overall displacement of the plastic zone boundary is relatively small.

In the above calculation, the initial stress field was not considered. For the mucky soil, the stress increase, $\sigma_{p} = C\cos \varphi$, which made the soil enter the plastic state very small. To satisfy the condition that soil easily enters the plastic state, the influence range of the plastic zone needs to be large so that the overall displacement of the plastic zone boundary can be considered relatively small and simplified as follows: $u_{p}^{2} \to 0$. The following equation was obtained:

15 $$1 + \Delta = 2u_{p} \frac{{R_{p} }}{{R_{u}^{2} }} + \frac{{R_{p}^{2} }}{{R_{u}^{2} }}\Delta .$$

For mucky soil, the pore water pressure was difficult to dissipate during the construction of the sand pile, the soil particles were uncompressible, and the amount of compression in the plastic zone was negligible, so the following equation was obtained:

16 $$1 = 2u_{p} \frac{{R_{p} }}{{R_{u}^{2} }}.$$

The following equation was obtained based on Eqs. (10), (11), and (16):

17 $$\frac{{R_{p} }}{{R_{u} }} = \sqrt {\frac{E}{2(1 + v)C\cos \varphi }} .$$

By substituting Eq. (17) into Eq. (13), the following equation was obtained:

18 $$p_{u} = C\left[ {(\cos \varphi + ctg\varphi )\left( {\frac{E}{2(1 + v)C\cos \varphi }} \right)^{{\frac{\sin \varphi }{{1 + \sin \varphi }}}} - ctg\varphi } \right].$$

The equation of stress and path can be obtained:

19 $$\sigma_{r} = C\left[ {(\cos \varphi + ctg\varphi )\left( {\frac{E}{2(1 + v)C\cos \varphi }} \right)^{{\frac{\sin \varphi }{{1 + \sin \varphi }}}} \left( {\frac{{R_{u} }}{r}} \right)^{{\frac{2\sin \varphi }{{1 + \sin \varphi }}}} - ctg\varphi } \right].$$

### Increase of additional stress induced by sand pile construction

When the sand piles are arranged in the shape of an equilateral triangle whose side length is $s$, interactions occur between the sand piles, where $d_{e}$ is the influence range of a single sand pile, and $r_{e}$ is the influence radius. It can be seen from Eq. (19) that the radial stress decreases with increased *r*. For safety considerations in engineering design, the increase in the additional stress of the soil around the pile caused by the cavity expansion of a single sand pile has a uniform distribution. The value is the additional stress at the boundary of the single pile; that is, the average additional stress increase in the entire plastic zone is $\sigma_{eq}$.

20 $$\sigma_{r} = C\left[ {(\cos \varphi + ctg\varphi )\left( {\frac{E}{2(1 + v)C\cos \varphi }} \right)^{{\frac{\sin \varphi }{{1 + \sin \varphi }}}} \left( {\frac{{R_{u} }}{{r_{e} }}} \right)^{{\frac{2\sin \varphi }{{1 + \sin \varphi }}}} - ctg\varphi } \right].$$

The following equation was obtained^16^:

21 $$d_{e} = 1.05s.$$

The replacement rate of the sand pile is represented by $m$, which can be expressed as follows:

22 $$m = \frac{{d^{2} }}{{d_{e}^{2} }} = \left( {\frac{{R_{u} }}{{1.05r_{e} }}} \right)^{2} .$$

By substituting Eq. (22) into Eq. (20), the following equation was obtained:

23 $$\sigma_{eq} = C\left[ {(\cos \varphi + ctg\varphi )\left( {\frac{0.551mE}{{(1 + v)C\cos \varphi }}} \right)^{{\frac{\sin \varphi }{{1 + \sin \varphi }}}} - ctg\varphi } \right].$$

## Increase of effective stress during the load phase

Open caisson foundations are often used in large-scale bridge foundation construction. Since the caisson is a temporary project during the pre-fabrication and sinking process, large settlement of the caisson or controllable inclination are allowed. Moreover, the construction of the caisson in the pre-fabrication process takes a relatively long time, and the additional stress is gradually converted into effective stress, so the improvement of the bearing capacity of the foundation cannot be ignored.

### Calculation of influence depth

The distribution of the additional stress of the foundation can be expressed by the contour line. If the additional stress $\sigma_{z} = 0.1p$, the influence of the additional stress is negligible, whereas for the strip load, the contour line of the additional stress passes below the center approximately^17^ when $z \approx 6B$. Therefore, the depth for the strip load is $H = 6B$.

### Calculation of degree of consolidation

The improved Jun Takagi method^16^ was used to calculate the degree of consolidation, and the equation is as follows:

24 $$\overline{{U_{t} }} = \sum\limits_{i = 1}^{n} {\frac{{\mathop {q_{i} }\limits^{ \cdot } }}{{\sum {\Delta p} }}} \left[ {(T_{i} - T_{i - 1} ) - \frac{\alpha }{\beta }e^{ - \beta t} (e^{{\beta T_{i} }} - e^{{\beta T_{i - 1} }} )} \right],$$

where $\overline{{U_{t} }}$ is the average degree of consolidation of the foundation within the depth range calculated within a given time of *t*, $\mathop {q_{i} }\limits^{ \cdot }$ (kPa/d) is the loading rate of the i-th load, $\sum {\Delta p}$ (kPa) is the accumulated value of the load at each level, $T_{i} ,T_{i - 1}$ (d) are the starting and ending times of the *i*-th load, and $\alpha ,\beta$ are the parameters of the consolidated drainage conditions of the foundation soil.

Parameters $\alpha ,\beta$ can be expressed as follows:

25 $$\alpha = \frac{8}{{\pi^{2} }}.$$

26 $$\beta = \frac{{8c_{h} }}{{F_{n} d_{e}^{2} }} + \frac{{\pi^{2} c_{v} }}{{4H^{2} }}.$$

27 $$n = \frac{{d_{e} }}{{d_{w} }}.$$

28 $$F_{n} = \frac{{n^{2} }}{{n^{2} - 1}}\ln (n) - \frac{{3n^{2} - 1}}{{4n^{2} }}.$$

### Relationship between effective stress and total stress during the sand pile construction

The relationship between the average effective stress of soil and total stress can be expressed by the following equation:

29 $$U = \frac{{p^{\prime}_{zt} }}{{p^{\prime}_{z\infty } }} = \frac{{p^{\prime}}}{p},$$

where $p^{\prime}_{zt}$($p^{\prime}$) represents the effective stress at time *t*, and $p^{\prime}_{z\infty }$($p$) stands for the effective stress at the time of complete consolidation.

30 $$p = \frac{{\sigma_{1} + \sigma_{2} + \sigma_{3} }}{3}.$$

The soil is isotropic, and the equation of calculating the static soil pressure is as follows:

31 $$\sigma_{1} = \gamma H + \sum {\Delta p_{z} } .$$

32 $$p_{0} = \sigma_{2} = \sigma_{3} = K_{0} \gamma H + \sigma_{eq} ,$$

where *H* is the buried depth, *K*_*0*_ is the static soil pressure coefficient, $p_{z}$ is the additional stress, and $\gamma$ is the dead load of the soil.

When no load was applied, or the applied load was not converted into effective stress, the following equation was given:

33 $$\sum {\Delta p}_{z} = 0.$$

34 $$\sigma_{eq} = 0.$$

35 $$p = \frac{{(1 + 2K_{0} )\gamma H}}{3}.$$

After sand pile construction was completed, the total stress with additional load applied could be expressed as follows:

36 $$p = \frac{{(1 + 2K_{0} )\gamma H + \sum {\Delta p_{z} } + 2\sigma_{eq} }}{3}.$$

## Relationship between effective stress and bearing capacity of the foundation

### Relationship between effective stress and water content

The groundwater level in the coastal area was relatively high and the stratum had been located below a stable water line for a long time. Thus, it could be assumed that the stratum is saturated, that is, $S_{r} = 1$, and the following equation was obtained:

37 $$S_{r} = \frac{{wG_{s} }}{e},$$

that is, $$e = wG_{s}$$.

For normal consolidated soil,

38 $$e_{0} - e + \lambda \ln p^{\prime} = 0.$$

Equation (38) can be changed to the following equation:

39 $$e_{0} - e + \lambda \ln Up = 0,$$

where $e$ is the reference void ratio, $e_{0}$ is the void ratio on the normal consolidation curve, $p^{\prime}$ represents the average effective stress, *U* denotes the consolidation degree, and $p$ denotes the average total stress.

### Relationship between bearing capacity of the foundation and water content

The relationship between the bearing capacity of a soft soil foundation and water content was given^18^ (Table 1).

**Table 1:** Basic bearing capacity of the soft soil foundation.

Natural water content *w* (%)	36	40	45	50	55	65	75
Basic bearing capacity *f*_0_ (kPa)	100	90	80	70	60	50	40

**Figure 3:**
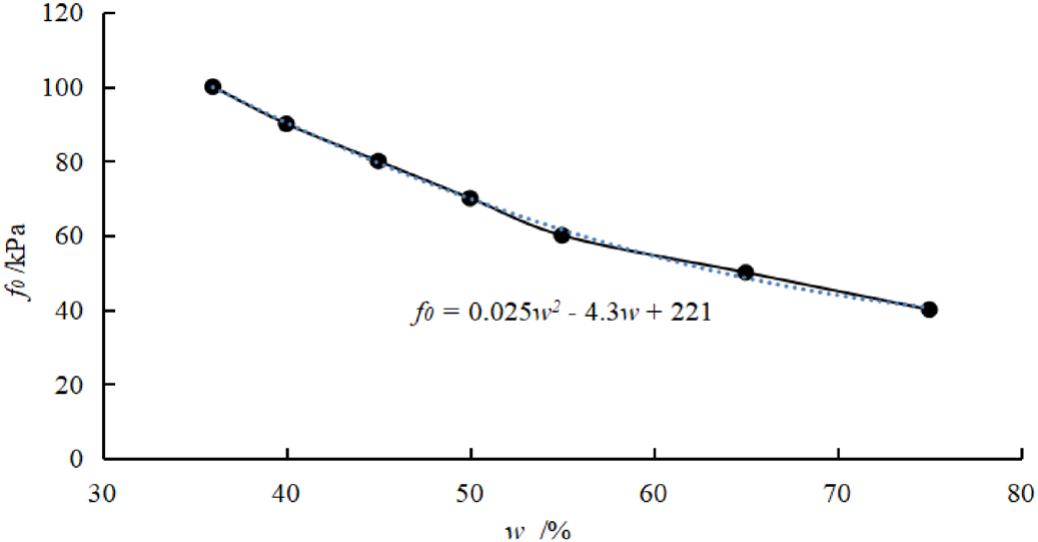
Curve of basic bearing capacity with natural water content.

The data in the above table is shown graphically, as shown in the figure below (Fig. 3).

The relationship between basic bearing capacity *f*_0_ and natural water content *w* is obtained:

40 $$f_{0} = 0.025w^{2} - 4.3w + 221,$$

The average effective stress increase can be obtained based on the external load, the size of the sand pile, and the buried depth of the soil layer, and, thereby, the change of the void ratio and the water content can be obtained. Since the water content of the soft soil foundation is well correlated with the bearing capacity of the foundation, the increase of the bearing capacity of the foundation can be obtained based on the water content, providing a basis for the design calculation of the sand pile composite foundation.

## Engineering example

The calculation method proposed in this study was applied to the south anchor foundation of Wenzhou Oujiang Estuary Bridge, which is the world's most thick mucky soil layer of super large land caisson. The caisson foundation was applied to the south anchor, the plane dimension of the caisson was 70 × 63 m, the total height of the caisson was 67.5 m, the standard wall thickness was 2.0 m, the partition wall was 1.2 m thick and 8 m high, the common partition wall was 6.1 m high, and thirty 10.84 × 10 m rectangular well holes were set. The caisson was located in a deep mucky soil layer with an average thickness of 36 m and a local thickness of 40 m.

### Geology and hydrogeology

Table 2 shows the simplification and parameters of the soil layer. As for the mucky clay in the surface layer (Number ②_1_), if the pre-consolidation pressure is greater than the gravity of the soil, the mucky clay should be judged as slightly over-consolidated soil, and the slope of the compression curve $k{ = }0.064$ can be directly used in the calculation, so the average effective pre-consolidation pressure is *P*_c_ = 85.6 kPa. If the average effective stress load is less than the average effective pre-consolidation pressure, the slope $\kappa$ should be used. When the average effective stress load is greater than the average effective pre-consolidation pressure, the slope $\lambda$ ought to be used.

**Table 2:** Physico-mechanical properties of caisson soil.

No	Soil layer	Thickness (m)	Pc (kPa)	$\lambda$	Compression modulus (MPa)	Permeability coefficient (10^−6^ cm/s)	Bearing capacity eigenvalue (kPa)	Side friction (kPa)
–	Sand bed	3	–	–	–	–	300	20
②_1_	Mucky clay	12.73	135.1	0.32	2.44	0.13	60	12
②_3_	Silt	15	91.27	0.58	1.93	0.22	55	10
③_3_	Mucky clay	5.22	150.8	0.40	2.18	0.40	60	15

The mechanical parameters of the soil layer are shown in Table 3.

**Table 3:** Physical parameters of the soil layer.

Layer no	Name	*w* (%)	*e*	G_s_	C_cu_ (kPa)	Φ_cu_ (°)	K_0_
②_1_	Mucky clay	47.2	1.312	2.72	16.3	14.9	0.45
②_3_	Silt	59.1	1.669	2.75	12.4	14	0.41
③_2_	Mucky clay	47.8	1.342	2.71	14.5	16.1	0.5

### Calculation of replacement rate of the sand pile

To simplify the calculation process, it was assumed that the excess pore pressure induced by the additional load had completely dissipated. The increase of the additional stress induced during the sand pile construction is inseparable from the replacement rate of the sand pile. The replacement rate can be preset and substituted into Eq. (23), and the replacement rate can be obtained. If the error between the preset replacement rate and the calculated replacement rate is less than 5%, the calculated replacement ratio is the acceptable replacement rate. In this study, the replacement rate was assumed to be *m* = 0.36.

**Figure 4:**
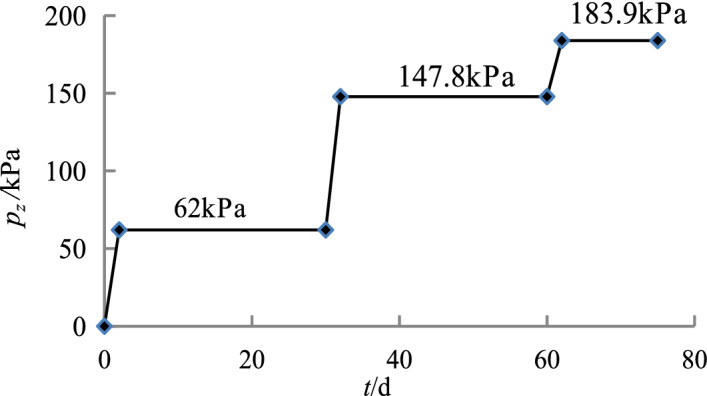
Loading process of the caisson in Sections 1–3.

**Figure 5:**
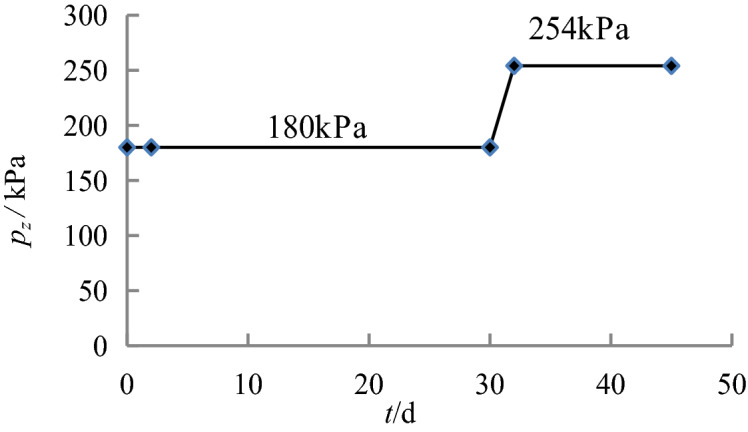
Loading process of the caisson in Sections 4–5.

**Figure 6:**
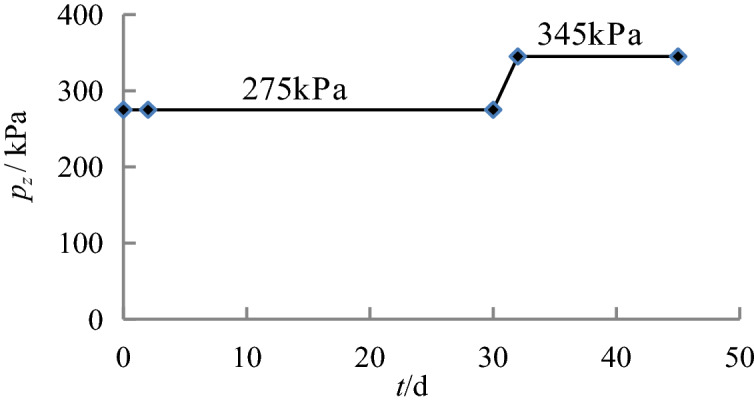
Loading process of the caisson in Sections 6–7.

**Figure 7:**
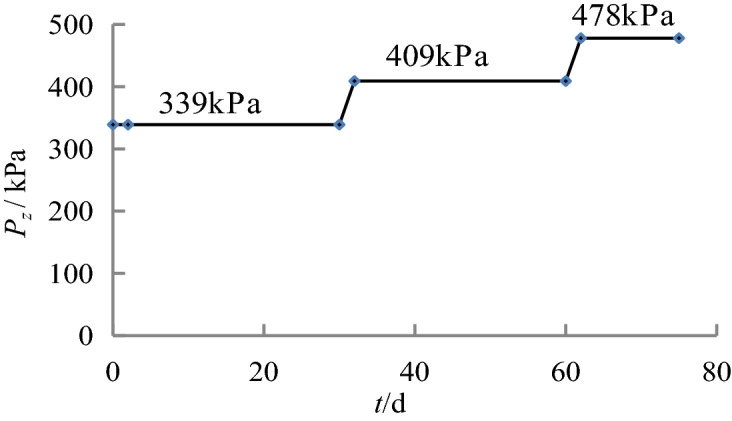
Loading process of the caisson Sections 8–10.

The relationships between the loads applied to the foundation and time are shown in Figs. 4, 5, 6 and 7.

Table 4 shows the calculation results.

**Table 4:** Change in water content after dissipation of pore pressure.

Loading stage	Original average effective ground stress (kPa)	Average effective ground stress after full consolidation of soil (kPa)	*e*	*w* (%)	Note
Sections 1–3 of the caisson	29.1	132.3	1.11	40.8	Original ground
Sections 4–5 of the caisson	31.7	153.9	1.06	39.0	Digging at a depth of 5 m with water injected
Sections 6–7 of the caisson	91	219.1	1.16	42.2	Digging at a depth of 15 m with water injected
Sections 8–10 of the caisson	167	322.4	1.08	40.4	Digging at a depth of 27 m with water injected

The bearing capacity of the foundation was calculated according to the water content (Table 5).

**Table 5:** Calculation of the replacement rate.

Loading stage	Bearing capacity eigenvalue of the foundation needed by the base (kPa)	Bearing capacity eigenvalue of the sand pile (kPa)	Bearing capacity eigenvalue calculated by considering the consolidation (kPa)	Replacement rate, *m*
Sections 1–3 of the caisson	144	322	88.4	0.24
Sections 4–5 of the caisson	88	322	92.5	–
Sections 6–7 of the caisson	115	246	85.6	0.23
Sections 8–10 of the caisson	160	287	89.2	0.36

The bearing capacity of the sand pile was expressed as follows^19^:

41 $$k = \frac{{20.8C_{u} }}{K},$$

where *C*_*u*_ represents cohesion, and *K* is the safety factor, which is recommended to be 1.05.

The equation of calculating the bearing capacity of the composite foundation^20^ is as follows:

42 $$m = \frac{{f_{spk} - f_{sk} }}{{f_{pk} - f_{sk} }}.$$

Then, the replacement rate of the sand pile was obtained, and the calculation results are shown in Table 5.

The calculated pile-soil stress ratio was between 2.8 and 3.6, which is in accordance with the pattern that the stress ratio of cohesive soil should be between 2 and 4 and the strength of undisturbed soil is high and the value is large^20^.

### Design of sand pile and verification of degree of consolidation

#### Design of sand pile

The replacement rate of the sand piles was set as *m* = 0.36, and the sand piles were arranged in the shape of a regular triangle. The diameter of each sand pile was 0.6 m, and the space between the sand piles was 0.95 m. The bearing capacity of the foundation, calculated according to Eq. (42), is shown in Table 6.

**Table 6:** Bearing capacity eigenvalue after foundation treatment.

Loading stage	Bearing capacity eigenvalue of the sand pile (kPa)	Bearing capacity eigenvalue calculated by considering the consolidation (kPa)	Replacement rate, *m*	Bearing capacity eigenvalue of the foundation (kPa)
Sections 1–3 of the caisson	322	88.4	0.36	172.5
Sections 4–5 of the caisson	322	92.5	0.36	175.1
Sections 6–7 of the caisson	246	85.6	0.36	143.3
Sections 8–10 of the caisson	287	89.2	0.36	160.4

#### Verification of degree of consolidation

Upon the completion of the sand pile construction, the additional stress was gradually converted into effective stress, and the pore pressure gradually disappeared. The degree of consolidation was calculated within the influence depth range of the additional stress. The results show that all the additional stress had been converted into effective stress before the caisson began to sink, and the degree of consolidation reached 100%.

### In-situ test

**Figure 8:**
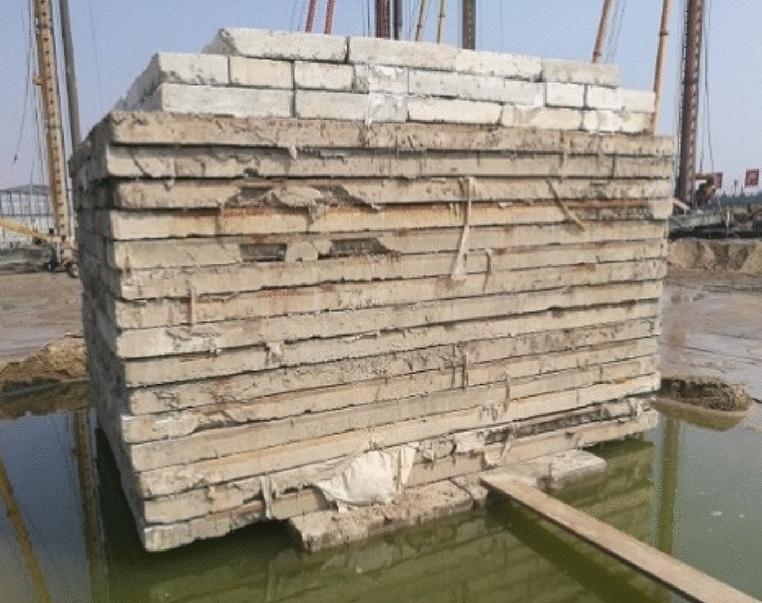
On-site preloading.

**Figure 9:**
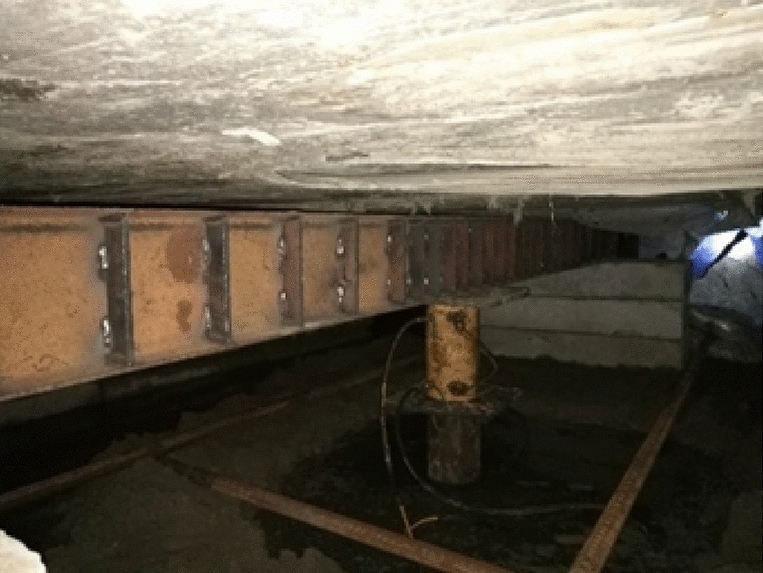
On-site plate load test.

The in-situ test was carried out at a randomly selected test point at the engineering site, and the sand pile was constructed per the replacement rate *m* = 0.36. Considering that the plate load test can only reflect the bearing capacity of the shallow foundation, the in-situ test can only verify the change of the bearing capacity of the foundation after the action of Sections 1–3 of the caisson. Therefore, a load of 180 kPa was applied to the composite foundation, and the bearing capacity of the composite foundation was tested after the soil pore pressure between the composite foundation piles had completely dissipated (see Figs. 8, 9).

**Figure 10:**
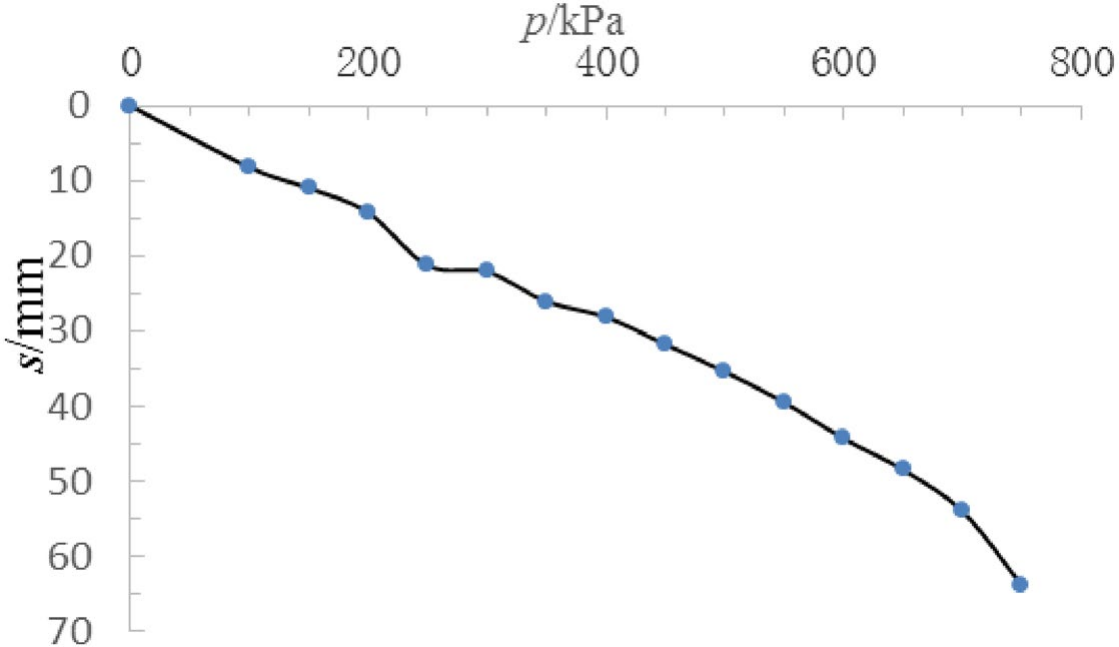
*p*–*s* curve of the composite foundation.

The loading plate had a diameter of 1000 mm, and the test was terminated^16^ once it was loaded to 60 mm. The bearing capacity eigenvalue of the foundation was determined according to the relative deformation amount^19^. For soil layers with high compressibility, the maximum value of relative deformation did not exceed 0.015, that is, when the settlement amount was 15 mm, the corresponding load was 205 kPa, which is larger than the design value of 172.5 kPa (see Fig. 10). This shows that the above calculation method not only makes rational use of the improvement of foundation bearing capacity caused by consolidation but also has a large security coefficient, which can provide reference for similar projects.

## Conclusions

In this study, the reasons why the bearing capacity of the sand pile composite foundation is much larger than the design value was explored. A feasible method of calculating the bearing capacity of the foundation was proposed based on the cavity expansion theory, the *e*–*p* curve theory, and empirical data. This method can optimize the design of sand pile composite foundations.

The main contributions of this study are listed as follows:

1. Cavity diameter expansion in sand pile construction was simplified into a planar problem, the cavity expansion theory was used to simplify the calculation of the increase of the horizontal stress of the soil between the piles caused by the sand compaction piles, and the expressions of the change in displacement rate and horizontal stress were established.
2. Considering the vertical effective stress increase caused by silt consolidation, the e–p curve was used to establish the relationship between the increase of the horizontal and vertical effective stress and the void ratio.
3. Based on the close relationship between the bearing capacity of the foundation in the mucky soil layer and the water content, the response of the bearing capacity of the foundation to the effective stress increase, the void ratio increase, and the water content increase was established. According to this relationship, it was determined that the increase of the effective stress caused an increase in the bearing capacity of the foundation.

For projects with long preloading or layered loading, under the premise of allowing large foundation deformation, the increased bearing capacity of the foundation caused by sand pile compaction construction and preloading can be fully considered to reduce the replacement rate and lower the construction cost.
